# Supplementary material for: Potential for Zika Virus to Establish a Sylvatic Transmission Cycle in the Americas
Source: PLoS Negl Trop Dis. 2016 Dec 15;10(12):e0005055. doi: 10.1371/journal.pntd.0005055 (PMC5157942; doi:10.1371/journal.pntd.0005055)
Supplement: S1 Text — (PDF) [file pntd.0005055.s001.pdf]

Supporting Information for Potential for Zika virus to establish a sylvatic transmission cycle in the Americas

Benjamin M. Althouse, Nikos Vasilakis, Amadou A. Sall, Mawlouth Diallo, Scott C. Weaver, Kathryn A. Hanley

Model description

The model is based on a previously published model of sylvatic dengue transmission [1, 2]. Briefly, primates are born susceptible to Zika virus (ZIKAV) at various rates, μ_{p_i} (proportional to the lifespan of the primate) and are infected by infectious mosquitoes at rate $r_{m_j p_i}\beta_{m_j p_i}(t)$ for mosquito species $1, \ldots, j$. Primates recover at rate γ_{p_i} and mosquitoes are infectious for their entire lifespan. Parameters are given in Table S1.

The transition rates for our stochastic model are below:

$$
\begin{aligned}
S_{m_j} \to S_{m_j} + 1 &= \mu_{m_j}(S_{m_j}(t) + (1-\rho)I_{m_j}(t)) && (1)\\
S_{m_j} \to S_{m_j} - 1 &= \nu_{m_j} S_{m_j} && (2)\\
\left.\begin{array}{r} S_{m_j} \to S_{m_j} - 1 \\ I_{m_j} \to I_{m_j} + 1 \end{array}\right\} &= \sum_i r_{m_j p_i}\beta_{p_i m_j}(t) I_{p_i} S_{m_j}/N_j && (3)\\
I_{m_j} \to I_{m_j} + 1 &= \rho I_{m_j} && (4)\\
I_{m_j} \to I_{m_j} - 1 &= \nu_{m_j} I_{m_j} && (5)\\
S_{p_i} \to S_{p_i} + 1 &= \mu_{p_i} N_{p_i} && (6)\\
S_{p_i} \to S_{p_i} - 1 &= \nu_{p_i} S_{p_i} && (7)\\
I_{p_i} \to I_{p_i} + 1 &= \iota N_{p_i} && (8)\\
\left.\begin{array}{r} S_{p_i} \to S_{p_i} - 1 \\ I_{p_i} \to I_{p_i} + 1 \end{array}\right\} &= \sum_j r_{m_j p_i}\beta_{m_j p_i}(t) I_{m_j} S_{p_i}/N_j && (9)\\
I_{p_i} \to I_{p_i} - 1 &= \nu_{p_i} I_{p_i} && (10)\\
\left.\begin{array}{r} R_{p_i} \to R_{p_i} + 1 \\ I_{p_i} \to I_{p_i} - 1 \end{array}\right\} &= \gamma_{p_i} I_{p_i} && (11)\\
R_{p_i} \to R_{p_i} - 1 &= \nu_{p_i} R_{p_i} && (12)\\
& && (13)
\end{aligned}
$$

with

$$\beta_{p_i m_j}(t) = b_{p_i m_j}[1 + c_j \cdot \cos(t * 2\pi/365)] \quad (14)$$

$$\beta_{m_j p_i}(t) = b_{m_j p_i}[1 + c_j \cdot \cos(t * 2\pi/365)] \quad (15)$$

$$N_{m_j} = S_{m_j} + I_{m_j} \quad (16)$$

$$N_{p_i} = S_{p_i} + I_{p_i} + R_{p_i} \quad (17)$$

$$N_j = \sum_i \left(\frac{r_{m_j p_i}}{\sum_i r_{m_j p_i}} \right) N_{p_i} \quad (18)$$

We run simulations with 1 infectious primate introduced into an entirely susceptible population. We examine the effects of primate and mosquito population size, primate birthrate (population turnover), and force of infection on the probability of ZIKV establishment. Simulations were run and we calculated the proportion of simulations not becoming extinct after introduction of a ZIKV infected host (ie, establishing a sylvatic cycle).

Population turnover

Key to calculating the probability of establishing Zika is the rate at which the host population produces susceptible individuals to maintain endemic infection levels. Using data from Ernest et al. [3] we examine the number of litters per primate per year by the birthrate, as well as the rate of population turnover (defined as the reciprocal of litters per year). Figure S1 shows relatively fast population turnover at birthrates explored in the model. Additionally we highlight primate species found in Brazil (green triangles).

References

[1] Althouse BM, Lessler J, Sall AA, Diallo M, Hanley KA, Watts DM, et al. Synchrony of sylvatic dengue isolations: a multi-host, multi-vector SIR model of dengue virus transmission in Senegal. PLoS Negl Trop Dis. 2012;6(11):e1928. doi:10.1371/journal.pntd.0001928.

[2] Althouse BM, Hanley KA. The tortoise or the hare? Impacts of within-host dynamics on transmission success of arthropod-borne viruses. Phil Trans R Soc B. 2015;370(1675):20140299.

[3] Ernest SM. Life history characteristics of placental nonvolant mammals: ecological archives E084-093. Ecology. 2003;84(12):3402–3402.
