# Supplementary material for: Potential for Zika Virus to Establish a Sylvatic Transmission Cycle in the Americas
Source: PLoS Negl Trop Dis. 2016 Dec 15;10(12):e0005055. doi: 10.1371/journal.pntd.0005055 (PMC5157942; doi:10.1371/journal.pntd.0005055)
Supplement: S1 Table — (PDF) [file pntd.0005055.s002.pdf]

Parameter	Description	Baseline Value
t	Time in days	
$r_{m_j p_i}$	Biting rate per day to primates by mosquito j [1, 2]	0.5 days^{-1}
$b_{p_i m_j}$	Baseline transmission probability, from primate i to mosquito j	0.3
$b_{m_j p_i}$	Baseline transmission probability, mosquito j to primate i [1, 3, 4]	0.3
c_j	Percent of the magnitude of seasonal variation for mosquito j	between $(0, 1)$, 0.05 unless otherwise specified
μ_{p_i}	primate birth rate (= 1/lifespan) [5]	$1/(15 * 365)$ to $1/(60 * 365)$ days^{-1}
ν_{p_i}	Primate death rate, set equal to birth rate	
γ_{p_i}	Primate recovery rate [6, 7, 8]	1/4 days^{-1}
μ_{m_j}	Mosquito j birth rate	1/7 days^{-1}
ν_{m_j}	Mosquito death rate, set equal to birth rate	
ρ	Mosquito rate of transovarial transmission	0
ι	Rate of infectious introduction	$0 - 10^{-4}$ years^{-1}

Table S1: **Model Parameters** Justifications for baseline values not given above are given in subsequent sections.

References

[1] Diallo M, Ba Y, Sall AA, Diop OM, Ndione JA, Mondo M, et al. Amplification of the sylvatic cycle of dengue virus type 2, Senegal, 1999-2000: entomologic findings and epidemiologic considerations. Emerg Infect Dis. 2003;9(3):362–7.

[2] Diallo M, Ba Y, Faye O, Soumare ML, Dia I, Sall AA. Vector competence of Aedes aegypti populations from Senegal for sylvatic and epidemic dengue 2 virus isolated in West Africa. Trans R Soc Trop Med Hyg. 2008;102(5):493–8. doi:10.1016/j.trstmh.2008.02.010.

[3] Diallo M, Sall AA, Moncayo AC, Ba Y, Fernandez Z, Ortiz D, et al. Potential role of sylvatic and domestic African mosquito species in dengue emergence. Am J Trop Med Hyg. 2005;73(2):445–9.

[4] Vasilakis N, Tesh RB, Weaver SC. Sylvatic dengue virus type 2 activity in humans, Nigeria, 1966. Emerg Infect Dis. 2008;14(3):502–4.

[5] Ernest SM. Life history characteristics of placental nonvolant mammals: ecological archives E084-093. Ecology. 2003;84(12):3402–3402.

[6] Gubler DJ, Suharyono W, Tan R, Abidin M, Sie A. Viraemia in patients with naturally acquired dengue infection. Bull World Health Organ. 1981;59(4):623–30.

[7] Vaughn DW, Green S, Kalayanarooj S, Innis BL, Nimmannitya S, Suntayakorn S, et al. Dengue in the early febrile phase: viremia and antibody responses. J Infect Dis. 1997;176(2):322–30.

[8] Vaughn DW, Green S, Kalayanarooj S, Innis BL, Nimmannitya S, Suntayakorn S, et al. Dengue viremia titer, antibody response pattern, and virus serotype correlate with disease severity. J Infect Dis. 2000;181(1):2–9. doi:10.1086/315215.
