# Supplementary figures and images for: Potential for Zika Virus to Establish a Sylvatic Transmission Cycle in the Americas
Source: PLoS Negl Trop Dis. 2016 Dec 15;10(12):e0005055. doi: 10.1371/journal.pntd.0005055 (PMC5157942; doi:10.1371/journal.pntd.0005055)

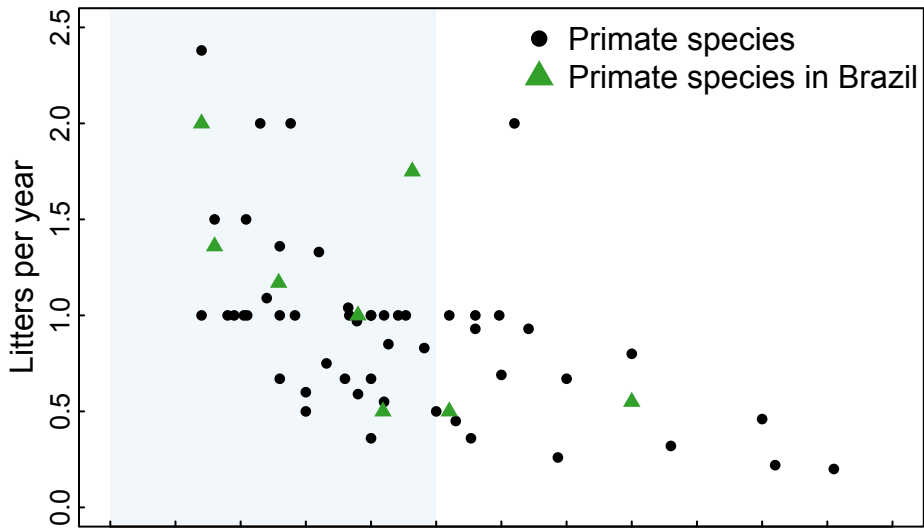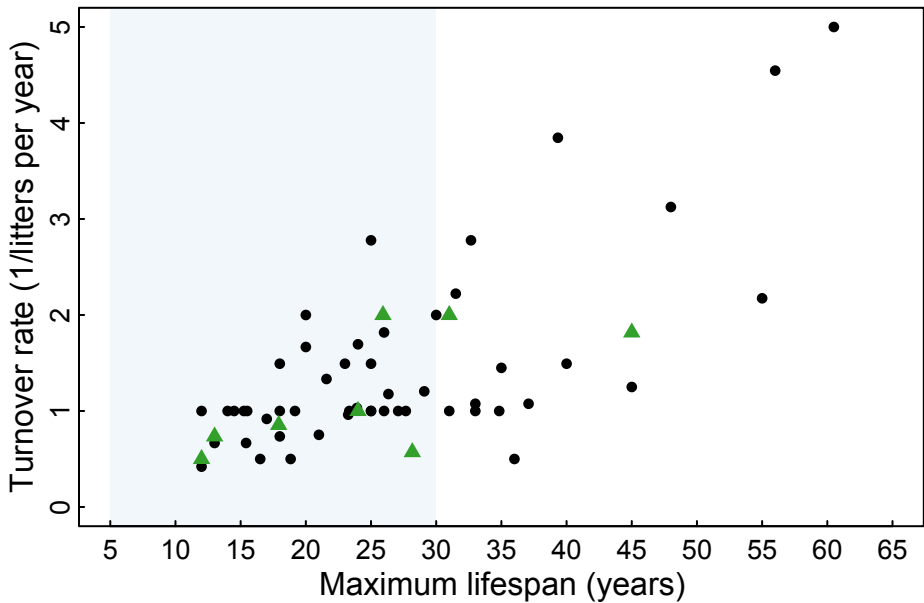

Supplement: S1 Fig — Figure shows the average number of litters per year and the turnover rate (1/litters per year) against maximum natural lifespan for 156 primate species. Those species found in Brazil are highlighted in green and include: Alouatta caraya, Alouatta seniculus, Aotus azarai, Aotus trivirgatus, Ateles belzebuth, Ateles paniscus, Brachyteles arachnoides, Cacajao calvus, Callicebus cupreus, Callimico goeldii, Callithrix flaviceps, Callithrix jacchus, Callithrix penicillata, Callithrix pygmaea, Cebus apella, Cebus olivaceus, Chiropotes albinasus, Chiropotes satanas, Lagothrix lagotricha, Leontopithecus rosalia, Pithecia monachus, Pithecia pithecia, Saguinus bicolor, Saguinus fuscicollis, Saguinus imperator, Saguinus labiatus, Saguinus midas, Saguinus mystax, Saguinus nigricollis. Data from Ernest et al. Life history characteristics of placental nonvolant mammals: ecological archives E084-093. Ecology. 2003;84(12):3402?3402. (PDF) [file pntd.0005055.s003.pdf]
